# Bispectral index-guided general anaesthesia in combination with interscalene block reduces desflurane consumption in arthroscopic shoulder surgery: a clinical comparison of bupivacaine versus levobupivacaine

**DOI:** 10.1186/s12871-015-0087-8

**Published:** 2015-07-21

**Authors:** Levent Ozturk, Elvin Kesimci, Tuna Albayrak, Orhan Kanbak

**Affiliations:** 1 Department of Anaesthesiology and Reanimation, Yıldırım Beyazıt University Ataturk Training and Research Hospital, Bilkent, Ankara, 06800 Turkey; 2 Anaesthesiology and Intensive Care Department, Ankara Ataturk Training and Research Hospital, Ankara, Turkey

**Keywords:** General anaesthesia, Interscalene block, Shoulder arthroscopy

## Abstract

**Background:**

The goal of this study was to compare the influence of an interscalene brachial plexus block (ISB) performed with either bupivacaine or levobupivacaine in conjunction with general anaesthesia (GA) on desflurane consumption, which was titrated to maintain the recovery profiles and postoperative analgesia while also keeping the bispectral index score (BIS) between 40 and 60 in patients undergoing arthroscopic shoulder surgery.

**Methods:**

Sixty patients undergoing arthroscopic shoulder surgery were prospectively randomized to receive GA with desflurane alone (group C) or in combination with a preoperative ISB by either bupivacaine 0.25 % (group B) 40 ml or levobupivacaine 0.25 % (group L) 40 ml. BIS scores or respiratory and hemodynamic parameters during the operation, recovery characteristics, consumed doses of desflurane and pain intensities were evaluated.

**Results:**

The eye opening time was 4.0 ± 2.5 minutes for group B, 4.6 ± 2.4 minutes for group L, and 6.2 ± 2.1 minutes for group C (*p* < 0.05). Group B and group L saved 36 % and 25 % desflurane per unit time respectively when compared with group C (*p* < 0.001and p < 0.05) while the mean pain scores and analgesic requirements the first day after surgery were higher in group C (*p* < 0.05).

**Conclusions:**

Because of lower desflurane consumption, a superior recovery profile, and a high degree of patient acceptance, general anaesthesia in combination with interscalene block may be preferred in arthroscopic shoulder surgery.

**Trial registration:**

The trial registration number is ACTRN12613000381785

## Background

It has been stated frequently that the combination of a regional nerve block with general anaesthesia (GA) may be beneficial, particularly in patients scheduled for major surgery [1]. Local anesthetics administered by different routes cause a decrease in the required doses and a decline in the use of hypnotic drugs in order to obtain a defined depth of anaesthesia [2]. For shoulder surgery, both GA and nerve block anaesthesia have been performed [3]. The combination of an interscalene brachial plexus block (ISB) and GA allows for safer control of respiration and easier maintenance of surgical analgesia. Although higher patient satisfaction with lower intraoperative and immediate postoperative narcotic requirements has been demonstrated when ISB is administered in combination with GA, controversy remains as to whether this combination alters anaesthetic depth and hypnotic anaesthetic requirements, thereby modifying recovery times [4, 5]. Bupivacaine is one of the most frequently used long-acting local anaesthetics. However, side effects related to high doses have limited its usefulness. Levobupivacaine is the S-enantiomer of bupivacaine, and it has been demonstrated to be equally effective [6]. Additionally, it is associated with less cardiac adverse effects owing to its lower affinity for cardiac sodium channels [7]. The clinical application of levobupivacaine and bupivacaine has been evaluated in many studies, but there is no clinical study which has compared desflurane’s sparing effect of ISB with bispectral index-guided GA.

The aim of this study was to evaluate this sparing effect performed by levobupivacaine or bupivacaine in patients undergoing arthroscopic shoulder surgery with GA. In addition, the recovery profile, postoperative pain, patient satisfaction, and adverse effects were also compared [3].

## Methods

Following ethics committee approval (Ethics Committee No:5 Ankara/Turkey), 60 patients classified as American Society of Anaesthesiolgists (ASA) I-IIwho were 18–65 years old and who underwent elective arthroscopic shoulder surgery by the same surgeon were included in this prospective, randomized, double-blind clinical trial. At the preoperative visit, details of the anaesthetic technique and study protocol were fully explained, and written consent was obtained from each patient prior to the study. Those who had general contraindications for ISB, obstructive pulmonary disease, diabetes, neuropathy, contralateral diaphragmatic paralysis, a history of allergic reaction to any of the study drugs, ongoing hypnotic therapy, or any documented preoperative systemic disease that could interfere with general anaesthesia were excluded from the study. None of the patients received premedication. In the operating room, an intravenous (IV) catheter was inserted on the non-operated side, and standard monitors were applied (GE Datex-Ohmeda S/5™ Anaesthesia Monitor, Helsinki, Finland). The baseline bispectral index score (BIS), heart rate (HR), noninvasive blood pressure, peripheral oxygen saturation (SpO2), and respiratory rate (RR) values were recorded and measured at predetermined time intervals throughout the surgery. From a list of random numbers, instructions for randomization were prepared in sealed envelopes for each patient before the start of the study. The patients were allocated into one of three groups in a double-blind manner. Group B (*n* = 20) received a single-shot ISB with bupivacaine 0.25 % (Marcaine, Astra Zeneca, Sweden) 40 ml before induction. Group L (*n* = 20) received a single-shot ISB with levobupivacaine 0.25 % (Chirocaine, Abbott Laboratories, North Chicago, USA) 40 ml before induction in combination with GA. Group C (*n* = 20) received GA alone. The nurse preparing and labeling the study drugs was blinded to the study procedures. In addition, the blocks and measurements throughout the study were performed by two different anesthetists who were also blinded to the treatment groups. The skin was cleaned with an antiseptic solution and 1 ml of lidocaine, of which 20 mg/ml was used for infiltration of the skin of the injection site subcutaneously. A standardized ISB technique was employed by following Winnie’s landmarks using a nerve stimulator and a 22-gauge x 50 mm stimulating needle (Stimuplex®; B. Braun, Melsungen AG, Germany) [8]. The initial current output of the nerve stimulator was set at 1 mA at 2 Hz. The interscalene groove was identified with the patient’s head turned to the side opposite to that being blocked. Skin puncture was performed, and the needle was advanced until a contraction of the deltoid or biceps muscle appeared [9]. The needle position was then adjusted until a twitch could still be elicited at a current output of less than 0.3 mA. After a negative aspiration test, the local anaesthetic was injected. Immediately after block placement, sensory block was assessed by pinprick at one minute intervals in the C4-6 dermatomes by a clinician unaware of the injected solution. After evidence of a successful sensory and motor block was obtained, the patients received a standardized anaesthetic protocol. Following administration of 100 % oxygen, anaesthesia was induced with IV thiopental 5–7 mg/ml and IV fentanyl 2 μg/kg. Then the patients received IV rocuronium 0.6 mg/kg, and the trachea was intubated so that the lungs were mechanically ventilated with a tidal volume of 8–10 ml/kg, with the ventilatory rate adjusted to maintain an end-tidal carbon dioxide concentration (partial pressure) of 30–35 mm Hg. Anaesthesia was continued with delivered (FD) desflurane 6 % (FD desflurane Suprane®, Baxter, Puerto Rico, USA) in 60 % nitrous oxide with oxygen, and the fresh gas flow was standardized. The desflurane concentration was then titrated to keep the BIS score in the 40–60 range. If the BIS value was less than 40 for more than 30 seconds, the FD desflurane was decreased by 25 %. If the BIS values exceeded 60 formore than 30 seconds, an ‘inhalation bolus of desflurane’ was administered [10]. The patients did not receive any additional fentanyl doses. Hypotension (a 20 % decrease in relation to the baseline value) and bradycardia (HR < 45 beats/min) were recorded. The hypotension was treated with IV fluid replacement or by a decrease in the desflurane concentration keeping the limits of BIS score in the 40–60 range. If these limits couldn’t be achieved by changes in desflurane concentrations, then it was’t altered any more and, IV ephedrine 3–6 mg was used if necessary. In cases of bradycardia, IV atropine 0.5-1 mg was administered. Intraoperative muscle relaxation was provided by administration of incremental doses of 0.2 – 0.3 mg/kg of rocuronium. Fifteen minutes before the expected end of surgery, the desflurane was reduced in all patients to facilitate rapid emergence from the anaesthesia. At the beginning of skin closure, both nitrous oxide and desflurane administration were interrupted. The fresh gas flow was increased to 6 l/min of pure oxygen at the end of skin closure, and the recovery period began. Tracheal extubation was not carried out until the patient had adequate spontaneous ventilation with a tidal volume > 4 ml/kg and responded to verbal commands. Residual neuromuscular blockade was reversed with atropine 15 μg/kg IV and neostigmine 40 μg/kg IV if necessary. Emergence from anaesthesia was assessed by measuring the time to spontaneous eye opening and tracheal extubation, the latter corresponding to the end of the recovery period. Anaesthetic gas consumption was measured each time by the same observer who was blinded to the groups to which the patients were assigned.

The desflurane was administered by a Sigma Alpha vaporizer (Penlon Limited, UK), and the amount used was measured in milliliters after completion of each surgical procedure by refilling the vaporizer, which initially had been completely full. After surgery, all patients were transferred to the postanaesthesia care unit (PACU), where the heart rate (HR), mean arterial pressure (MAP) and respiratory rate (RR) were monitored. Side effects, such as hypotension, nausea, vomiting, hoarseness, Horner’s syndrome, and dyspnea, were documented. Patient satisfaction (0 = not satisfied, 1 = moderate, 2 = good, 3 = very good) and postoperative pain scores [using a 10 cm Visual Analog Scale (VAS) in which 0 cm = no pain and 10 cm = the worst pain imaginable] were evaluated on arrival at the PACU and at two, four, six, eight, and 24 hours after surgery. The duration of analgesia (time to first requested analgesic) was recorded, and the supplemental postoperative analgesia was standardized. If the VAS was ≥ 3, patients received 75 mg of intramuscular (IM) diclofenac followed by 50–100 mg of IV tramadol if the VAS remained unchanged after 30 minutes. Patients were discharged from the PACU according to the Aldrete discharge criteria [11].

### Statistical analysis

The primary end point of this study was defined as a reduction in desflurane consumption. Sample size estimation was performed by using MINITAB 15 software. Sample size was predetermined by using a power analysis: α = 0.05 and β = 0.2, and this showed that 19 patients per group would be sufficient. Data analysis was performed using the SPSS version 11.5 software program (SPSS Inc., Chicago, Illinois, USA). The Kolmogorov-Smirnov test was used to test the normality of distribution for continuous variables. The data was expressed as the number of patients and mean ± standard deviation (SD) (minimum-maximum), where applicable. For parametrical data, one way analysis of variance (ANOVA) was used, and the Bonferroni correction was applied when there was a significant difference. The repeated hemodynamic parameters and VAS were analyzed by repeated measures ANOVAwith Bonferroni adjustment for multiple comparisons. For gender, analgesic need, patient satisfaction, side effects, and ASA, the chi-square or Fisher’s exact tests were used. Statistical significance was set at a *p* value <0.05 for all analyses and *p* < 0.033 (0.1/3) for those that underwent Bonferroni adjustment.

## Results

Sixty patients were enrolled in this study with each group containing 20 patients. The groups were similar with respect to age, weight, height, ASA physical status, and duration of surgery and anaesthesia (Table 1).

**Table 1:** Patient characteristics and duration of surgery

	Group L	Group B	Group C	*p* value
	(*n* = 20)	(*n* = 20)	(*n* = 20)	
Age (yr)	48.1 ± 11.4	45.8 ± 13.3	39.2 ± 14.5	0.092
Sex (M/F)	11 (55) / 9 (45)	7 (35) / 13 (65)	12 (60) / 8 (40)	0.247
BMI	25.4 ± 1.8	25.8 ± 2.9	24.9 ± 2.0	0.489
ASA physical status I/II	12 (60) / 8 (40)	13 (65) / 7 (35)	15 (75) / 5 (25)	0.592
Duration of surgery (min)	99.1 ± 47.9	69.6 ± 32	89.6 ± 51.8	0.114
Duration of anaesthesia (min)	123.5 ± 50.4	88.8 ± 36.3	106.1 ± 51.5	0.053

Values are mean (SD) or n (%)

**Fig. 1:**
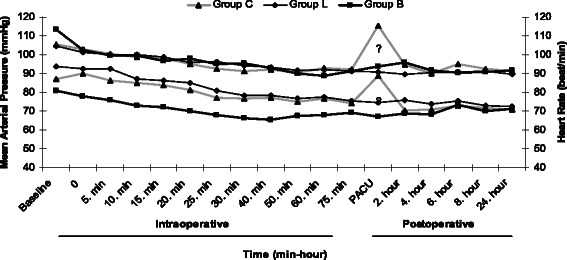
Perioperative mean arterial pressures and heart rate of patients. ? p<0.05: Compared with Group B and L

There were no significant differences among the groups in terms of the BIS scores or respiratory and hemodynamic parameters during the operation (*p* > 0.05). However, the HR and MAP values were significantly higher in group C on arrival at PACU compared with the other two groups (*p* < 0.05) (Fig. 1). The recovery period parameters were not significantly different between groups B and L. The time to eye opening was 4.0 ± 2.5 minutes for group B, 4.6 ± 2.4 minutes for group L, and 6.2 ± 2.1 minutes for group C (*p* < 0.05). The times to tracheal extubation were significantly shorter for groups B and L than for group C (*p* < 0.05) (Table 2). The cumulative consumed doses of desflurane were significantly less in group B (92.4 ± 43.7 mL), and this group saved 36 % and 15 % desflurane per unit time respectively compared with groups C and L (*p* < 0.05). Group L saved 25 % desflurane per unit time compared with group C (*p* > 0.05). The analgesic duration was significantly longer in group B than in groups L and C (*p* < 0.05). Similarly, a significance was also distinguished between groups L and C (*p* < 0.05) (Table 3).

**Table 2:** Recovery period parameters after termination of desflurane

	Group L	Group B	Group C	*p* value
	(*n* = 20)	(*n* = 20)	(*n* = 20)	
Open eyes (min)	4.6 ± 2.4	4.0 ± 2.5*	6.2 ± 2.1	0.012
Tracheal extubation (min)	1.8 ± 1.3*	2.0 ± 1.8*	4.0 ± 1.8	<0.000

Values are mean (SD)

*p < 0.05; compared with Group C

**Table 3:** Duration of analgesia, total amount of desfluran consumption, the cost of desflurane in each group

	Group L	Group B	Group C	*p* value
	(*n* = 20)	(*n* = 20)	(*n* = 20)	
Duration of analgesia (min)	962.2 ± 170.9*^,**^ (665–1245)	1181.5 ± 198.2*(748–1545)	122.6 ± 52.9 (58–229)	<0.000
Desflurane consumed (mL)	152.4 ± 64.2^**^ (40–470)	92.4 ± 43.7* (37.33-186.66)	175.1 ± 94.7 (80–390)	0.002
Volume of Desflurane per time unit(ml)	1.23 ± 0.23^**^ (0.53-1.57)	1.05 ± 0.25* (0.70-1.62)	1.63 ± 0.31 (1.14-2.31)	0.001

*p < 0.05: compared with Group C

**p < 0.05: compared with Group B

**Fig. 2:**
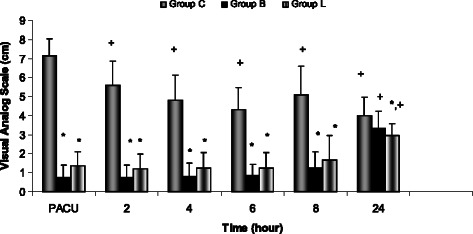
VAS pain scores (0 ± 10 cm) in the three groups during the first 24 h after operation. Values are mean (SD). *p<0.05: compared with Group C. +p<0.05: compared with VAS at PACU

The postoperative pain scores were significantly lower on arrival to PACU, and at two, four, six, and eight hoursr after surgery in group B and also at 24 hours in group L compared with group C (*P* < 0.05) (Fig. 2). Postoperatively, 95 % and 100 % of patients requested diclofenac as the first pain medication at 24 hours in group L and group B respectively, but this ratio was 100 % on arrival to PACU in group C (*p* < 0.000). Moreover, additional rescue tramadol was administered in 55 % of these patients (*p* < 0.000). Patient satisfaction was rated as “very good” in 75 % of the patients in group L and 65 % of those in group B (*p* < 0.05).

## Discussion

The results of this study demonstrated that an ISB by either bupivacaine or levobupivacaine in combination with GA reduced the total desflurane required to achieve atarget BIS of between 40 and 60 when compared with GA alone. A prolonged time until the first analgesic requirement and an earlier discharge were evident in both combined anaesthesia groups. Recently, the combination of GA with a regional nerve block has found wide acceptance due to adequate intraoperative pain control, alleviation of intraoperative surgical stress response and drug requirements, rapid recovery, adequate postoperative analgesia, and timely discharge [12, 13]. The effects of different types of epidural administration and concentrations of different local anaesthetics and opioids with respect to volatile anaesthetic use have been broadly investigated. Panousis et al. demonstrated that epidural administration of a high concentration of ropivacaine led to a significant sparing effect on desflurane concentration [14]. In another study, it was suggested that when combining GA with epidural anaesthesia, the amount of volatile anaesthetics required to achieve an appropriate anaesthetic depth were reduced to levels less than the expected values [15]. In this study, we primarily investigated the effects that two different preoperatively-administered local anaesthetics for ISB had on the required total volume of a volatile anaesthetic needed to ensure an adequate anaesthetic depth under GA by BIS monitorization. To our knowledge, our data is not directly comparable with any other study since there has been no other study comparing desflurane use and the recovery profile regarding ISB combined with GA and GA alone in patients undergoing arthroscopic shoulder surgery. It has been shown that the BIS is a reliable monitor of the depth of anaesthesia and desflurane requirements during general anaesthesia [16]. In another study, Song et al. assessed the consumption of sevoflurane and desflurane in patients undergoing tubal ligation with BIS monitorization and reported 30-38 % less use of volatile agents [17]. In the present study, among the three groups, we targeted a BIS score 10 between 40 and 60 to keep the same depth of anaesthesia. Since BIS monitoring was only evaluated for desflurane, no additional fentanyl was given after tracheal intubation. We not only saved desflurane consumption significantly but also increased the speed of recovery after the operation by adding the BIS to our standard monitorization. However, the durations of anaesthesia were different among the groups, though these were not significant. Due to this difference, we also calculated the consumption of desflurane per unit time.

Recent studies have indicated that GA is frequently associated with more postoperative pain, increased nausea and vomiting, less ability to void, and significant increases in the length of PACU stay for shoulder surgery [18, 19]. Moreover, some authors have found perceived disadvantages of GA versus ISB. In a study, Jochum et al. failed to demonstrate any superiority when using a combination of GA with ISB versus using ISB alone [20]. Similarly, Chelly et al. demonstrated that ISB alone was safe and effective and could contribute to shortening the duration of hospital stays [21]. In contrast, Grossi et al. reported that patients who were awake while undergoing surgery by supplementing ISB with GA experienced less stress [22]. In our study, the effects of desflurane were terminated as soon as possible because of this agent’s quick wash-out. This resulted in a modest reduction of end-tidal desflurane concentrations in patients at the end of surgery, which led to a significant reduction in recovery times. One item that hospitals must consider is the increased cost of anaesthesia during surgery and the role that plays in total hospital costs. Many different anaesthetic regimens may be used for cost savings in operating rooms. The shorter recovery variables of this study demonstrated the ability of an interscalene block, when combined with GA, to significantly reduce anaesthetic consumption and shorten the length of stay at PACU, which can result in cost savings.

In the present study, we used the same volumes and concentrations of levobupivacaine and bupivacaine to induce the block. Only a few studies exist which compare the efficacy of different local anaesthetics in cervical blocks. Recent studies in epidural analgesia have demonstrated that levobupivacaine has the identical potency of racemic bupivacaine [23]. Similar to the findings in the study by Lyons et al., many of the investigators suggested a comparable anaesthetic potency between these two agents [24, 25]. However, in this study, bupivacaine seemed to be a more potent agent than levobupivacaine in terms of desflurane use and duration of analgesia. A possible explanation for this difference might be the coincidental longer duration of surgery in group L.

Although this did not cause a significant difference between the two block groups in terms of duration of surgery, the desflurane consumed in group L was signicantly more than that used in group B. Thus, this result can not be interpreted in favor of the superiority of bupivacaine to levobupivaine with regard to local anaesthetic potency. The quality of recovery and all the VAS assessments were comparable between the two study groups, except for the VAS at the 24th hour postoperatively which was significantly lower in the levobupivacaine group. The quality and duration of analgesia provided by levobupivacaine was better than bupivacaine. The patients both consumed less rescue analgesics and complained less pain in levobupivacaine group at 24 hour. This may be due to the different profile of nerve block resolution of these two agents. These patients consumed no rescue analgesics until the block wore off. Surgical procedures of the shoulder are often performed with the patient in a semi-sitting position that can result in decreased ventricular volume and cardiac output due to pooling of blood in the lower extremities [26]. Additionally, hypotension and bradycardia due to an inhibitory reflex arising from cardiac sensory receptors with vagal afferents may occur in this position under ISB. In the study by Ozzeybek et al., although patients received ISB combined with GA, arterial blood pressure decreased significantly when the patients were tilted, despite adequate fluid administration and slow positioning of the patient [27]. In our study, the hemodynamics remained unchanged in all patients throughout the study period. No hypotensive episodes needed treatment in either group, probably because our study population was 18–65 years old and otherwise healthy except for being ASA I or II. Despite the many surgical advantages of this position, sedation and continous observation of the airway and hemodynamics which are of anaesthetic relevance may be difficult in awake patients [28]. It is known that inadequate sedation in these patients might result in complaints of noise or discomfort from prolonged immobility and can increase the risk of side effects unless there is further improvement in the analgesia quality [20]. We found no statistically significant differences with regard to the adverse effects among the groups in our study, but three patients in group L and four patients in group B developed Horner’s syndrome.

The study has several limitations. We couldn’t measure actual blood concentrations of bupivacaine or levobupivacaine, so despite the fact that identical anaesthetic dosages were administered, it is not possible to conclude that equal anesthetic effects were achieved. Second, this study should have been investigated in another group of patients with comorbidities and older age group (>70 years of age) to report about safety and early recovery.

## Conclusion

In conclusion, a preoperative interscalene block with either levobupivacaine or bupivacaine provided superior pain control for the first 16 hours after surgery, and the combination of ISB and GA also provided a safer control of respiration. This type of anaesthesia is a practical choice for patients undergoing arthroscopic shoulder surgery based on the lower requirements for postoperative analgesics, reduced desflurane consumption, and the possibility of cost reduction. Because of the low incidence of side effects, the lack of complications, and the high degree of patient acceptance, we recommend general anaesthesia in combination with interscalene block for patients undergoing shoulder surgery. Although the two long-acting local anaesthetics used in this study had similar pain scores, duration of analgesia, side effects, and patient satisfaction during the postoperative period, it should be kept in mind that levobupivacaine is associated with having a more reduced depressant effect on cardiovascular function than bupivacaine. Further studies of the effects of lower amounts of short-acting local anaesthetics in combination with GA are needed before final recommendations can be made.
